# A machine learning-based gene signature of response to the novel alkylating agent LP-184 distinguishes its potential tumor indications

**DOI:** 10.1186/s12859-021-04040-8

**Published:** 2021-03-02

**Authors:** Umesh Kathad, Aditya Kulkarni, Joseph Ryan McDermott, Jordan Wegner, Peter Carr, Neha Biyani, Rama Modali, Jean-Philippe Richard, Panna Sharma, Kishor Bhatia

**Affiliations:** 1 Lantern Pharma, Inc., 1920 McKinney Ave, 7th floor, Dallas, TX 75201 USA; 2 REPROCELL USA Inc., 9000 Virginia Manor Rd, Ste 207, Beltsville, MD 20705 USA

**Keywords:** Acylfulvene, LP-184, PTGR1, Cancer, Machine learning, Gene signature, Response prediction, Biomarker

## Abstract

**Background:**

Non-targeted cytotoxics with anticancer activity are often developed through preclinical stages using response criteria observed in cell lines and xenografts. A panel of the NCI-60 cell lines is frequently the first line to define tumor types that are optimally responsive. Open data on the gene expression of the NCI-60 cell lines, provides a unique opportunity to add another dimension to the preclinical development of such drugs by interrogating correlations with gene expression patterns. Machine learning can be used to reduce the complexity of whole genome gene expression patterns to derive manageable signatures of response. Application of machine learning in early phases of preclinical development is likely to allow a better positioning and ultimate clinical success of molecules. LP-184 is a highly potent novel alkylating agent where the preclinical development is being guided by a dedicated machine learning-derived response signature. We show the feasibility and the accuracy of such a signature of response by accurately predicting the response to LP-184 validated using wet lab derived IC50s on a panel of cell lines.

**Results:**

We applied our proprietary RADR® platform to an NCI-60 discovery dataset encompassing LP-184 IC50s and publicly available gene expression data. We used multiple feature selection layers followed by the XGBoost regression model and reduced the complexity of 20,000 gene expression values to generate a 16-gene signature leading to the identification of a set of predictive candidate biomarkers which form an LP-184 response gene signature. We further validated this signature and predicted response to an additional panel of cell lines. Considering fold change differences and correlation between actual and predicted LP-184 IC50 values as validation performance measures, we obtained 86% accuracy at four-fold cut-off, and a strong (r = 0.70) and significant (p value 1.36e−06) correlation between actual and predicted LP-184 sensitivity. In agreement with the perceived mechanism of action of LP-184, PTGR1 emerged as the top weighted gene.

**Conclusion:**

Integration of a machine learning-derived signature of response with in vitro assessment of LP-184 efficacy facilitated the derivation of manageable yet robust biomarkers which can be used to predict drug sensitivity with high accuracy and clinical value.

## Background

Chemotherapeutic drugs target cancer-specific characteristics, such as alterations in DNA repair, which has complicated roles in oncogenesis and tumor progression. Illudins are a class of sesquiterpene secondary metabolites initially researched in the 1950s due to potent cancer cytotoxicity, particularly illudins S and M [1]. Early investigations into illudin S revealed activity in tumors with multidrug resistance but were halted due to findings of high non-tumor toxicity [2, 3]. The poor therapeutic index of illudins led to development of semisynthetic alternatives, such as acylfulvenes [4]. Compared to illudins, acylfulvenes have better tumor-specific activity, and are particularly effective in tumors having deficiencies in DNA repair [5].

Mechanisms of illudin and acylfulvene cytotoxicity include DNA alkylation, resulting in cell-cycle arrest and apoptosis [6–9], generation of reactive oxygen species and the chemical modification of various intracellular proteins [10] as well as inhibition of cytosolic redox-regulating thiol-containing proteins such as glutathione reductase, thioredoxin reductase, and thioredoxin [11]. Unlike illudin S, acylfulvene tumor specificity appears to be based on a tumor-selective activation through reductive mechanisms that are mediated by enzymes such as Prostaglandin reductase 1 (PTGR1), or leukotriene B4 12-hydroxydehydrogenase [12]. Activated acylfulvenes can oxidize various cellular thiols, as well as create DNA adducts that disrupt DNA and RNA synthesis. Resolution of these adducts specifically requires transcription-coupled DNA repair, such as transcription-coupled nucleotide excision repair (TC-NER) [5, 13]. Tumors vary widely in their capacities to launch functional DNA repair, raising the idea that acylfulvenes would perform favorably if matched to TC-NER-deficient tumors.

In addition to key mechanistic knowledge on tumor specificity related to acylfulvene activation by PTGR1 and tumor resistance determined by TC-NER pathway components, there remain additional pathways known to be associated with their activity. We are therefore not fully aware of all possible mechanisms of action in tumor types under consideration for treatment with acylfulvenes, which may impede our capability to leverage this mechanistic knowledge into ultimate therapeutic decisions. An additional barrier remains in determining precisely how raw mechanistic knowledge is eventually translated into a useful measurement guiding therapeutic choices. Promising classes of compounds have not been further studied in specific subsets of cancers due to lack of a drug-tumor interaction knowledge base. These challenges will be more pronounced with novel acylfulvene derivatives that, while possibly showing appealing therapeutic indices, lack extensive mechanistic studies. A solution to the dual problems of having unknown determinants of therapeutic potential, and untested predictive value with currently known mechanisms, is the development of a signature designed to make these predictions using machine learning approaches. Machine learning approaches for gene signature development save significant amount of time from preliminary data generation to clinical application with rapid iterations allowing for hypothesis building and testing.

A gene signature can be used to predict expected outcomes of different patients or predict the likelihood of a successful therapeutic response with a given drug. With regard to cancer, development of a signature is possible through use of a combination of clinical features, gene expression, genomic alterations, and other data types. A gene signature can be similar to a biomarker in its characteristics, and, in some cases, a signature may be regarded as a larger set of biomarkers. Like a biomarker, a signature aims to have robust and reliable prognostic value, but both the methods used for derivation of a gene signature, and for computing its predictive power, will often differ. While traditional biomarkers might be based on extensive study and expert analysis with a cancer type, machine learning is ideally suited to the task of uncovering features that can successfully predict outcomes of interest, using various data sources. Data on mutations and gene expression are often used to develop signatures, and several studies have concluded that the mRNA expression signature of a tumor often has the most predictive power. With the recent expansion of high-quality databases in cancer and the reduced cost of doing whole-genome analyses in patients, there is great potential for improving accuracy and utility of gene signatures used in clinical predictions. With potential acylfulvene derivative therapies, machine learning methods have potential to identify the tumor characteristics that may be missed in human studies, and provide better predictions of the tumors where they will be therapeutically effective. Traditional gene signature of drug response could take several years after millions of dollars of laboratory resources, and may even fail to have clinical significance. Early integration of machine learning is critical to compress the time, risk and cost of exploring and achieving optimal positioning of compounds in cancers that can be particularly sensitive.

LP-184 is a next generation acylfulvene (AF) drug class member related to Illudin S, a toxin that occurs naturally in certain mushrooms [1, 14]. The chemical nomenclature of LP-184 is defined as *N*-hydroxy-*N*-(methylacylfulvene)urea. LP-184 is an alkylating prodrug that damages DNA and has broad antitumor activity that is preferentially activated in cancer cells expressing certain biomarkers. Another acylfulvene analog, Irofulven (hydroxymethylacylfulvene) [15], has been tested in multiple preclinical studies and clinical trials, both as monotherapy, and in combination with a variety of other chemotherapeutic agents [16–18]. Irofulven has previously evidenced clinically acceptable safety and tolerability. In preliminary studies, LP-184 efficacy has been established in a spectrum of various tumor cell lines, including the NCI-60 cell line panel.

Applications of machine-learning to genomic analysis in cancer have covered diagnosis, prognosis, and therapeutic response predictions [19–25]. Early efforts using multiple genes to form a signature employed traditional differential gene expression analysis (DGE), where genes that are most significantly differentially expressed (or have the greatest fold change) between groups that respond differently to therapy, or differ in prognosis, are identified in a standard approach [26]. In recent years, well established machine learning methods such as multiple factor analysis (MFA), support vector machines (SVMs), random forests, and others, have evolved and been newly applied in combination with cloud infrastructure to the same data and trained with corresponding information on the cancer-related outcomes of interest [27–32]. The data available for such training includes patient data, but this is often limited in quantity, and is also impacted by the heterogeneity of patient genetic backgrounds and other confounding factors. A popular alternative that resolves some of the issues with sample number and heterogeneity is to use cell line sensitivity as a surrogate for the response to chemotherapeutics, which is measured by the dose required to lower cell-growth by half (an IC50 value).

In this paper we describe the leveraging of LP-184 sensitivity and cancer cell line gene expression from the NCI-60 panel as training data to establish a set of candidate biomarkers forming an LP-184-specific gene signature predictive of response in solid tumors. Using RADR® (Response Algorithm for Drug positioning and Rescue), we implemented an optimized set and sequence of gene feature filtering and machine learning algorithms to derive a gene expression-based signature of response to LP-184. This signature was further validated in vitro and in silico in independent testing datasets, demonstrating its predictive value in identifying suitable tumor models as well as sensitive tumor types for advanced experimental validation and clinical translation. Notably, the signature includes the anticipated candidate biomarker PTGR1 that is highly correlated with LP-184 sensitivity and implicated in the induction of bioactivation of LP-184 which provides a strong biological rationale for clinical application. Further, our machine learning approach is able to rank the relative variable importance of each of the signature genes which would be useful in developing a scalable clinical companion diagnostic assay associated with LP-184 response. Using machine learning, starting from a pool of 18,000 genes, we have a reduced set of only 16 genes forming an LP-184 response signature. This provides a manageable biomarker panel as a basis for patient stratification and clinical advancement of LP-184.

## Results

### Derivation of LP-184 sensitivity signatures

**Fig. 1:**
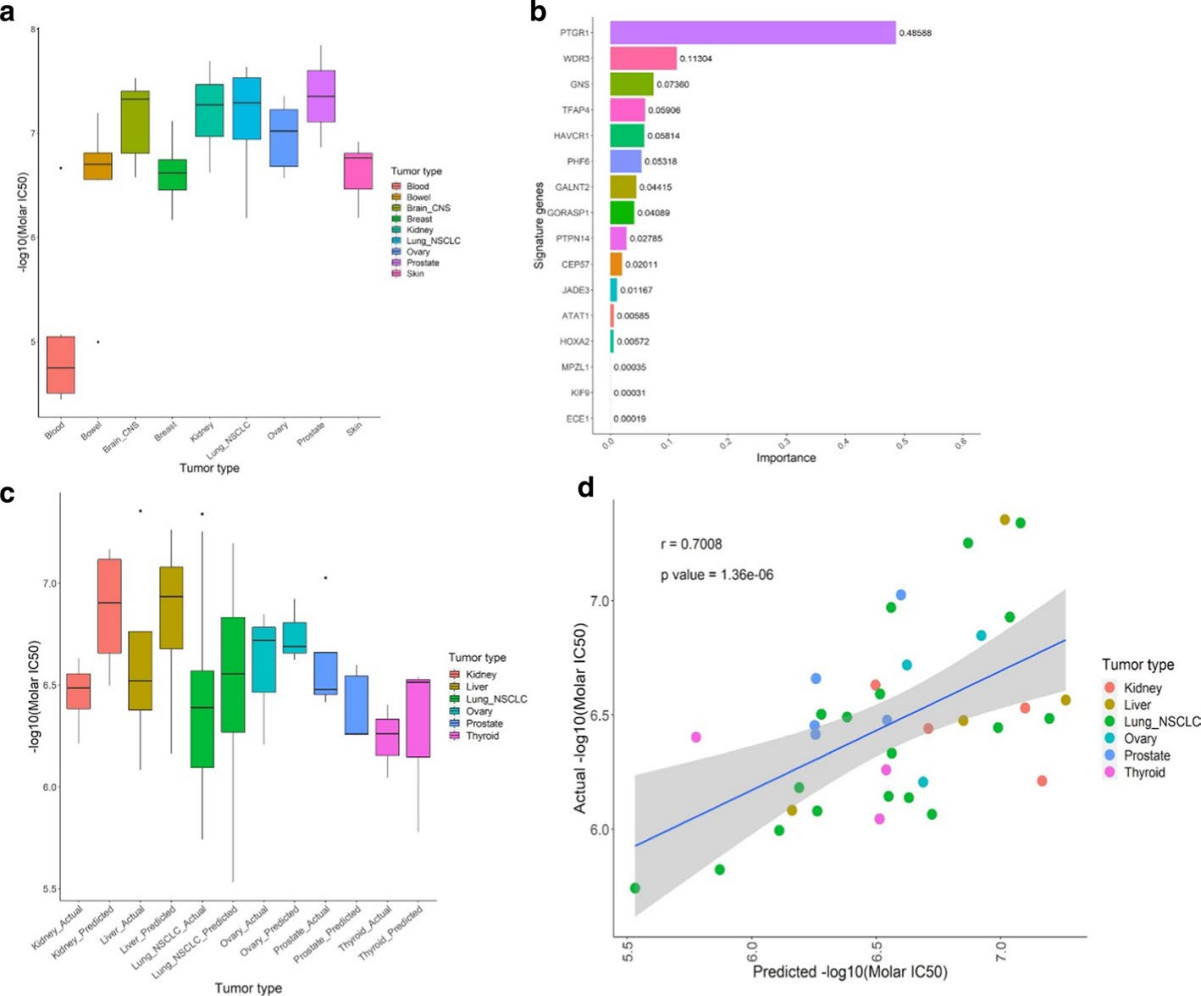
Comparison between actual and predicted LP-184 IC50 values. **a** Sensitivity profile of LP-184 in the 52 cell lines grouped by tumor type from NCI-60 panel. **b** Gene signature predictive of LP-184 response. **c **This boxplot shows proximity between actual and predicted LP-184 IC50 values (− log10 (Molar IC50)) on Y axis for individual tumor types on X axis from the blind test set of 37 solid tumor cell lines. **d** This graph shows Pearson correlation between actual and predicted LP-184 IC50 values from the blind test set of 37 solid tumor cell lines, covering 6 tumor types

To investigate whether a gene-expression signature in untreated tumors can predict sensitivity to LP-184, we retrieved microarray (23,059 transcripts) and RNA-seq (23,829 transcripts) data from 59 cancer cell lines in the NCI-60 database [33]. Using these cell lines, we directly assessed sensitivity through cell growth measurements 48 h after LP-184 exposure to calculate the IC_50_ (Fig. 1a). We observed a range of sensitivity for most tumor types, except in blood-derived cancers, which we therefore excluded from model building. The remaining 52 solid tumor lines used IC50 values as a training target and gene expression as input features. List of 52 cell lines with experimental IC50 (Molar) is given as Additional file 4: Table S2.

After splitting the training data into internal cross-validation training and test sets composed of 41 and 11 records respectively, we compared application of a number of machine-learning algorithms for their accuracy of predicting IC_50_ values (as a regression), or a sensitivity classification. Gene input either used all genes, or a subset of genes determined by a Pearson’s correlation threshold, which was further filtered by selecting subsets from Pathway enrichment analysis or gene-network analysis (described in Methods). We compared a range of machine-learning approaches including deep learning neural networks, decision trees, and other classical methods, with each performed as regressions and/ or classifications, where possible. We compared support vector machine (SVM), elastic net regression (ENR), random forest (RF), XGBoost, and deep neural networks, alone or in combination (Additional file 2: Figure S2). Using a Boruta feature selection (random forest based) [34], followed by XGBoost to refine selected features (removing genes one by one and building models using 5-time repeated tenfold cross validation), showed the lowest root mean squared error (RMSE), and greatest accuracy in LP-184 response prediction on the test set. Initial training used microarray data, which was found to generally perform better than RNA-seq data, although both datasets were highly correlated. We therefore chose a final model from the Boruta-XGBoost methods based on microarray data that provided a 16-gene signature predictive of LP-184 sensitivity (Fig. 1b).

### Evaluation of 16-gene model robustness

We tested whether predictions would perform similarly in non-NCI-60 cancer cell lines, so we selected 37 solid-tumor lines from the Cancer Cell Line Encyclopedia (CCLE) that covered those having either the low- or high-end of PTGR1 expression in microarray data. Using expression data from CCLE, our model predicted sensitivity of these cell lines, and, as shown in Fig. 1c and Fig. 1d, the predicted sensitivity was highly correlated to the experimentally-derived LP-184 sensitivity.

**Fig. 2:**
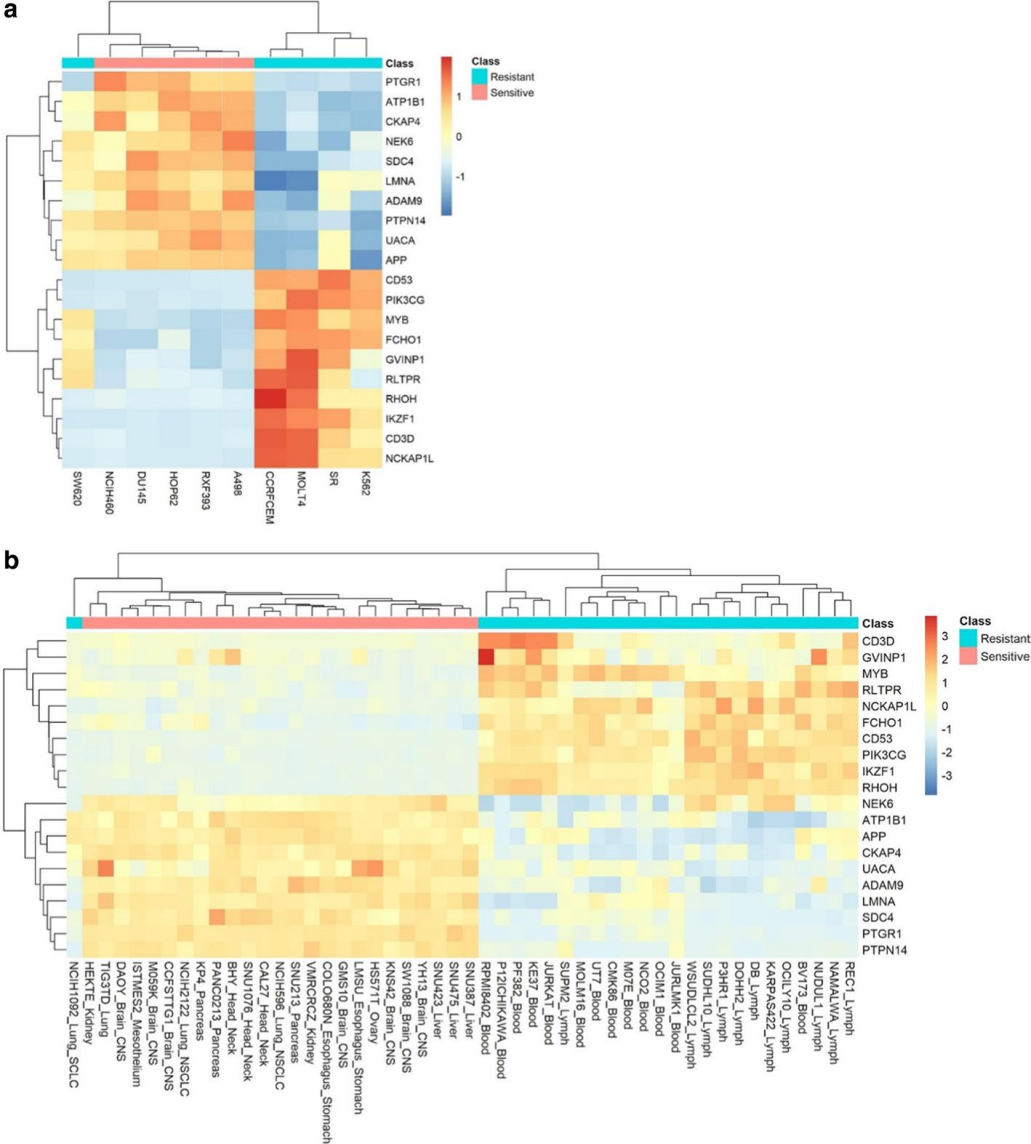
Co-clustering between genes highly correlated with LP-184 sensitivity in training set and cell lines subgroups. **a** This heat map displays the clustering pattern across the top 5 cell lines known to be sensitive and resistant to LP-184 (from actual IC50 in NCI-60 data) arranged in vertical columns and the top 20 correlated genes (10 positively correlated and 10 negatively correlated) with actual known LP-184 sensitivity arranged in horizontal rows. **b** This heat map displays the clustering pattern across the top 25 cell lines predicted sensitive and resistant to LP-184 (from predicted IC50 in CCLE testing data) arranged in vertical columns and the top 20 correlated genes (10 positively correlated and 10 negatively correlated) with actual known LP-184 sensitivity arranged in horizontal rows

We further analyzed the relationship of gene expression and the signature to LP-184 activity. Hierarchical clustering of top 5 known sensitive and top 5 known resistant cell lines from the NCI-60 panel clearly divided the cells (with one exception) between sensitive and resistant, and an obvious pattern with the top-20 most correlated genes emerged (Fig. 2a). Consistently, clustering of the most correlated genes to sensitivity in the CCLE test set showed all-but-one cell line was automatically grouped in the correct category by performing clustering with the same top-20 sensitivity-correlated genes across the top 25 predicted sensitive and top 25 predicted resistant cell lines (Fig. 2b). This indicated that both true LP-184 sensitivity in 58 NCI-60 lines and the signature-based predicted sensitivity in all 1000 CCLE lines (Additional file 3: Table S1) were robustly correlated to the same gene expression pattern, which is expected to be a causal association in some cases.

**Fig. 3:**
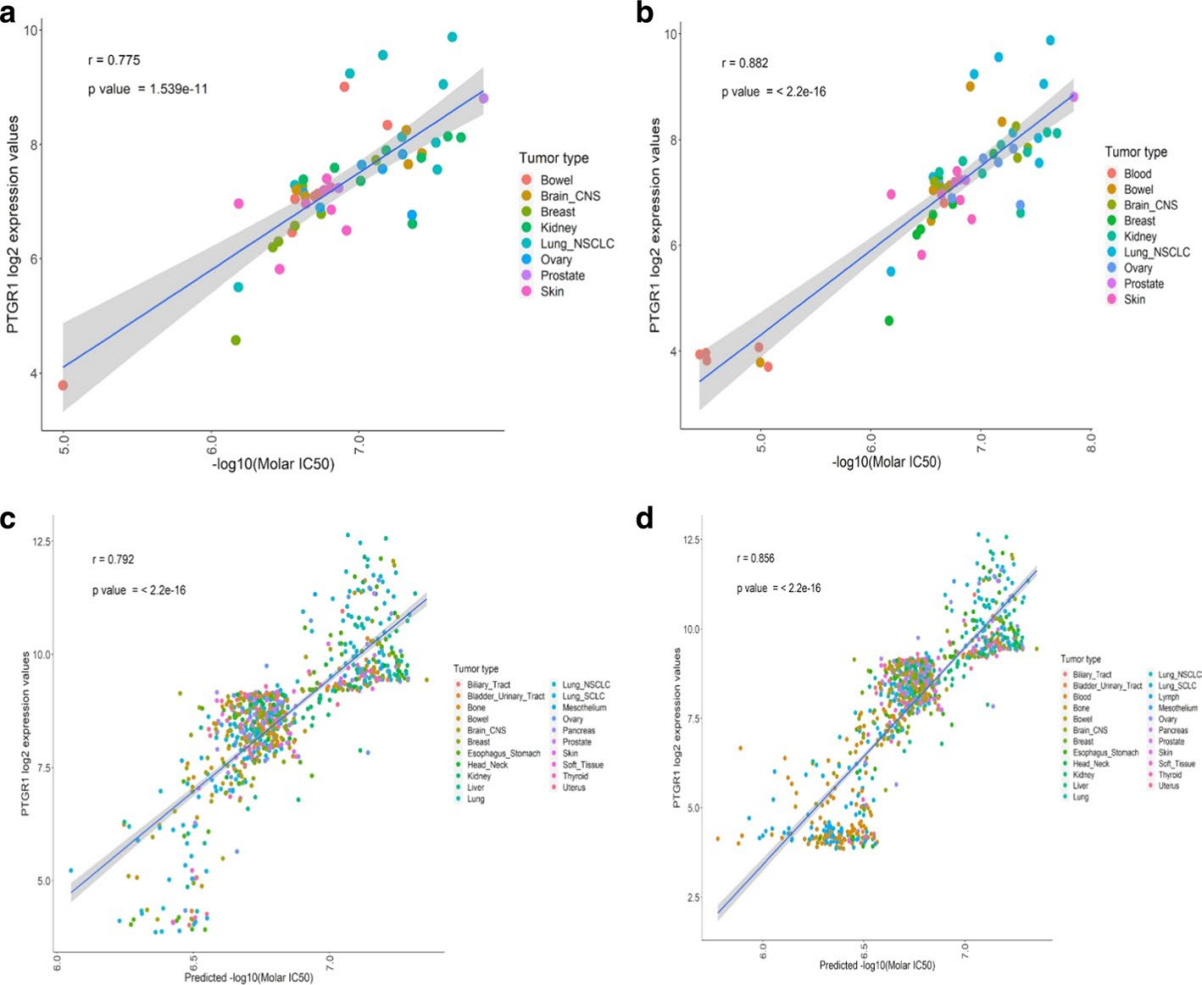
Correlation between PTGR1 transcript level (Y axis) and observed or predicted LP-184 IC50s (X axis). **a** PTGR1 gene expression correlation with actual IC50 from NCI-60 solid tumor cell lines (excluding blood) PTGR1 expression correlation from 52 NCI-60 solid tumor cell lines with LP184 drug sensitivity shows strong and significant (p value 1.539e−11) Pearson correlation (correlation coefficient r = 0.775). **b** PTGR1 gene expression correlation with actual IC50 from all NCI-60 cancer cell lines (including blood). **c** PTGR1 gene expression correlation with predicted IC50 from CCLE solid tumor cell lines (excluding blood and lymph). **d** PTGR1 gene expression correlation with predicted IC50 from all CCLE cell lines (including blood and lymph)

PTGR1 has been demonstrated to be a causal, critical determinant in the cytotoxicity of acylfulvenes through reductive activation mechanisms [12, 35, 36]. Across different parameters used in model building, we consistently found PTGR1 selected as the most important variable for predictions. Further, in our final model, PTGR1 alone represents almost half of the total variable importance among all 16 genes. The Pearson correlation coefficient (r) of PTGR1 expression with LP-184 sensitivity in the 52 solid tumors used in training was 0.775 (*p* value 1.539e−11) (Fig. 3a). If including blood, this showed a stronger and more significant association (r = 0.882 and *p* < 2.2e−16) (Fig. 3b). Likewise, there was high PTGR1 correlation to predicted sensitivity in 820 CCLE lines (all lines excluding those derived from blood; r = 0.792, and *p* < 2.2e−16), and the full 1000 lines of the CCLE (r = 0.856, and *p* < 2.2e−16) (Fig. 3c, d). This indicated our model’s expression signature correctly captured a known key causal factor underlying acylfulvene toxicity mechanism of action.

### Robust gene expression patterns in sensitive and resistant cancers

**Fig. 4:**
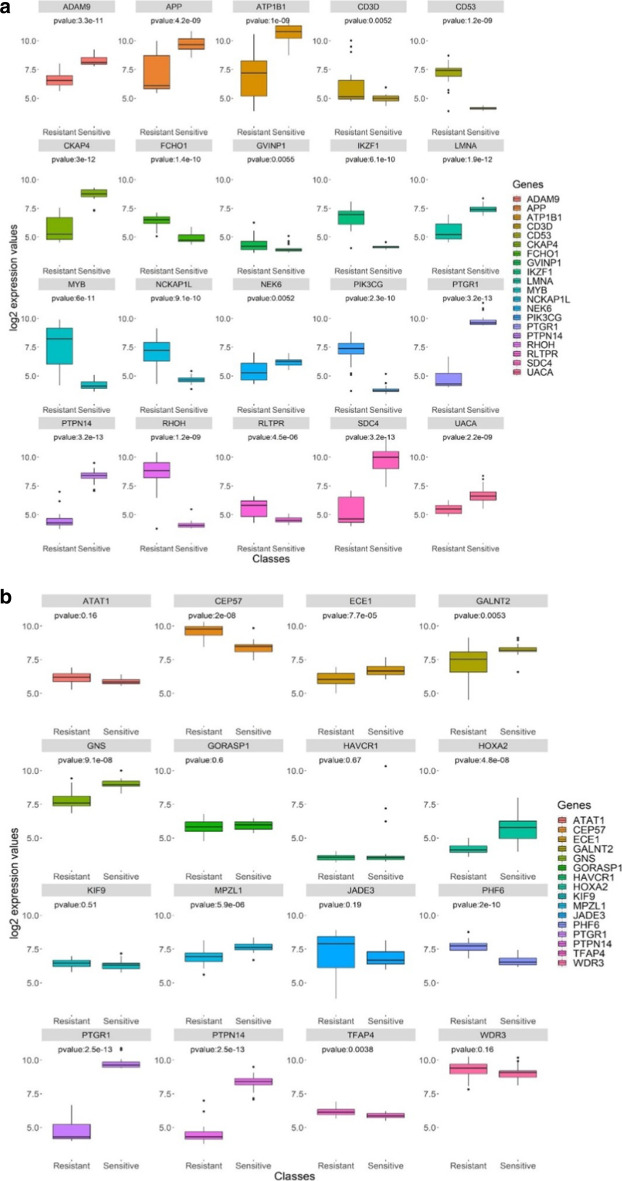
Classifying cancer cell lines into predicted LP-184 sensitivity groups based on gene expression levels. **a** This panel of box plots shows the differential expression of each of the top 20 correlated genes (10 positively correlated and 10 negatively correlated) with actual known LP-184 sensitivity in NCI-60 training data across the top 25 predicted sensitive and top 25 predicted resistant cell lines in the CCLE testing data. **b** This panel of box plots shows the differential expression of each of the 16 signature genes of LP-184 response across the top 25 predicted sensitive and top 25 predicted resistant cell lines in the CCLE testing data

We further looked at the association of the top-20 sensitivity correlates and signature genes, comparing differential expression in the top-25 ranked cell lines classified by either predicted sensitivity and resistance, which corresponds to cell lines having a range of IC50 from 14.3 nM to 35.9 μM, respectively. Individually looking at expression of the top-20 sensitivity gene correlates, we observed a stark contrast between groups. Each gene was significantly differentially expressed in resistant and sensitive lines using a Wilcoxon test (Fig. 4a). PTGR1 had a mean increase of ~ 2.5 log-scaled expression (*p* < 3.2e−13). Highly significant differences were also observed with PTPN14 (*p* < 3.2e−13), SDC4 (*p* < 3.2e−13), CKAP4 (*p* < 3e−12), ADAM9 (*p* < 3.3e−11), and PIK3CG (*p* < 2.3 e−10). Some of these may have novel roles in relation to cancer and/or the LP-184 mechanism of action, while some have been previously reported to play roles in cancer malignancy – e.g., PIK3CG which is tightly associated with growth and migration in several cancers.

We next sought to examine the comparative differential expression values of genes in the 16-gene model signature. The majority had distinct and significant differential expression between CCLE-predicted sensitive and resistant lines (Fig. 4b). As indicated in Fig. 4a, PTGR1 and PTPN14 had *p* < 3.2e−13, while other genes had substantially lower significance in differential expression comparisons. Five of the 16 genes (JADE3, KIF9, GORASP1, ATAT1 and WDR3) had *p* values > 0.05. This might indicate that performance of the predictive model on the blind set of 37 CCLE lines (Fig. 1c, d) was accurate despite these non-significant expression differences with these genes. However, as the predictive model does not rely on a direct linear conversion of expression values from the gene signature (instead being a decision-tree-based model), this may instead be evidence that the value of such genes by themselves has a more complex—potentially context dependent—relationship with predictions.

### Gene pathways enriched with LP-184-sensitivity pattern

**Fig. 5:**
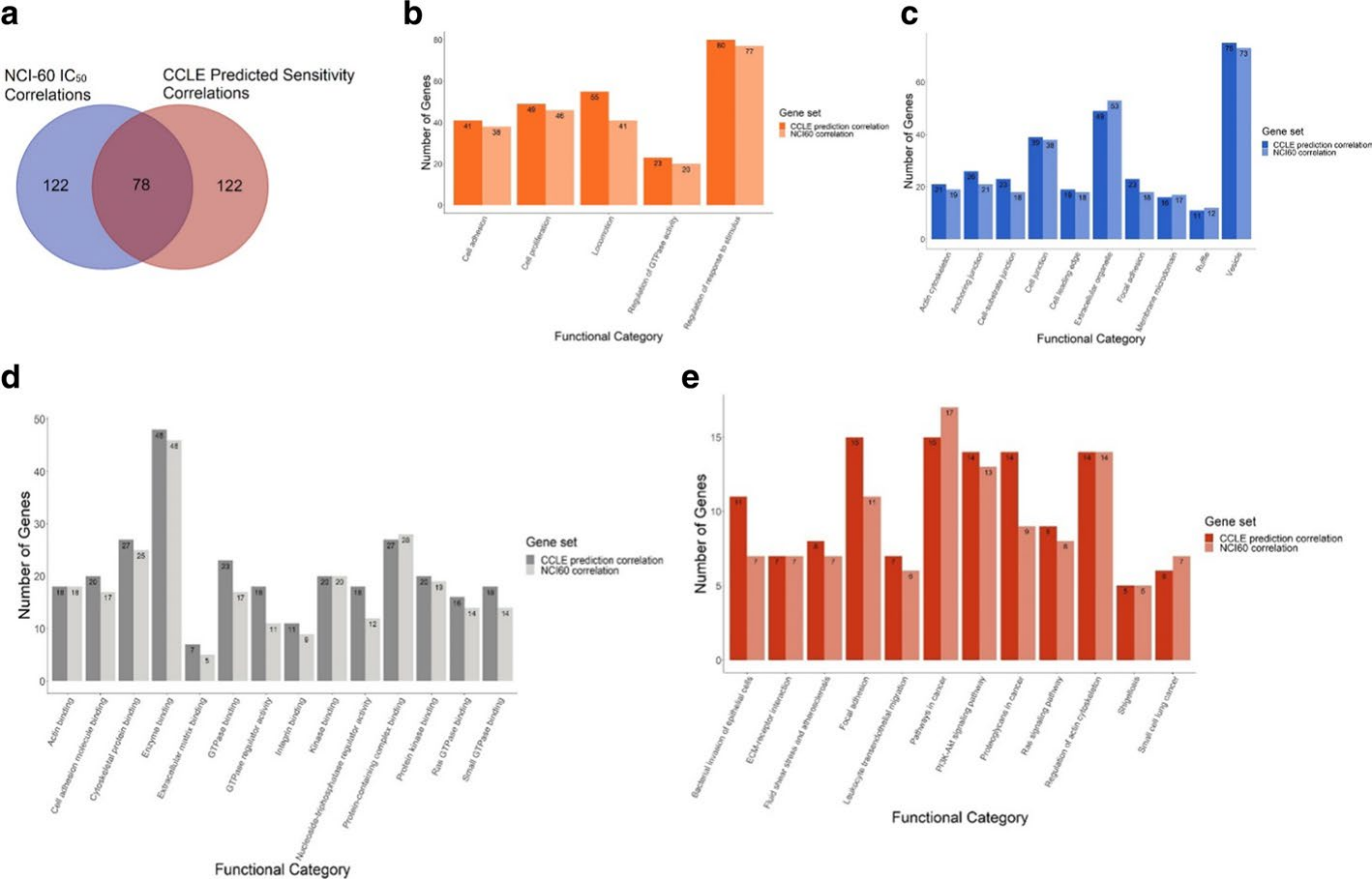
Overlap between and functional enrichment of LP-184 correlated genes from training and test sets. **a** This Venn diagram shows common genes between NCI-60 correlation with actual LP-184 sensitivity, and CCLE correlation with predicted LP-184 sensitivity. Bar charts show common GO enriched categories from **b** biological process, **c** cellular component, **d** molecular function aspects and **e** signaling pathway from KEGG (Kyoto Encyclopedia of Genes and Genomes), between NCI-60 top correlated genes and CCLE top correlated genes. Top 20 significant (FDR <  = 0.05) GO enrichments were selected for comparison

It is not clear whether chemotherapeutic efficacy occurred due to random variance in the expression of key genes, or whether there was an underlying expression pattern that related to cellular functions. To investigate functional relationships that sensitivity-correlated genes may have, we performed gene enrichment analysis with the top-100 positively and negatively correlated genes of both the gene correlations from actual LP-184 sensitivity in 58 NCI-60 cell lines, and predicted sensitivity of 1000 CCLE cell lines. We found that 39% of both were identical (Fig. 5a). We performed gene enrichment analysis in the separate groups, and examined pathways having significant enrichment and an FDR ≤ 0.05 cut off. A striking similarity between both sets’ pathway enrichment membership was observed (Fig. 5b-d). For example, approximately 90% of the total number of genes significantly enriched in the CCLE-prediction-correlation set for in the GO biological processes were also enriched in the NCI-60-correlation set (Fig. 5b). Similar overlaps were found in GO cellular component, GO molecular function, and KEGG signaling pathway enrichment numbers. Detailed gene list for each enrichment category is given in the Additional file 5: Table S3 & Additional file 6: Table S4.

Enriched pathways are apparently interrelated with regard to roles in cancer, involving the extracellular matrix, focal adhesion, the cytoskeleton, and Ras signaling. The activity of these pathways may provide a further marker for LP-184 sensitivity, and these associations are potentially causal. Our results collectively show that LP-184 IC50 values correlate to a robust gene pattern that can also be readily detected in the gene expression of numerous cancer lines by correlating their gene expression to the prediction of sensitivity derived from the 16-gene model. Not only is a consistent pattern identified, but the pattern has clear enrichment for particular cancer-related pathways.

### Tissue-dependent cancer sensitivity to LP-184

**Fig. 6:**
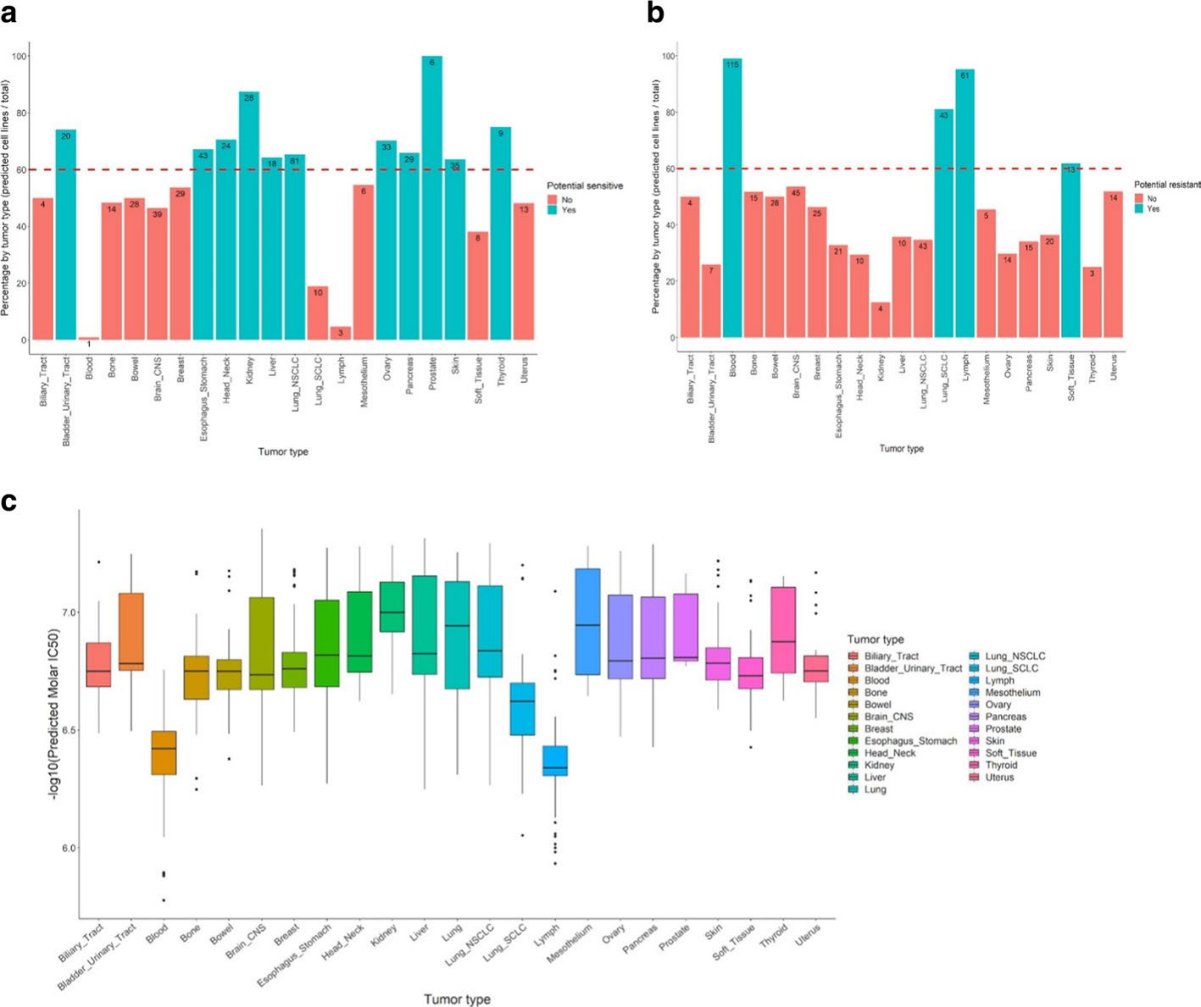
Potential LP-184 sensitive and resistant tumor types from the test set predictions. **a** This bar chart shows tumor types potentially sensitive to LP-184, highlighted in green. Y axis represents the percentage of cell lines by tumor type predicted as sensitive to LP-184, and X axis shows various tumor types. **b** This bar chart shows tumor types potentially resistant to LP-184, highlighted in red. Y axis represents the percentage of cell lines by tumor type predicted as resistant to LP-184, and X axis shows various tumor types. **c** This box plot shows the spread of predicted LP-184 IC50 values across multiple tumor types represented in the CCLE

The model for LP-184 sensitivity was used to determine whether and where LP-184 will be most effective, through using the expression values of the 16-gene signature to predict sensitivity in diverse cell types. Although we observe there is intra-tumor-type diversity in LP-184 sensitivity, and that this can be correctly predicted, some tumor types may be more likely to express the signature genes in the appropriate pattern. We compared percentages of predicted sensitive or resistant proportions in 23 cancer types from CCLE data, by first defining sensitive as having an IC50 z-score over 0, and resistant as those with z-scores below zero. This resulted in 484 cells being classified as sensitive and 516 as resistant. After excluding four unclassified lung cancer cell lines from further analysis, cancer types were categorized as a sensitive class if having > 60% of the cell lines within that cancer type as predicted sensitive, or conversely, as resistant if having > 60% of the cell lines within that cancer type as predicted resistant (Fig. 6ab). Bladder, esophageal, head and neck, kidney, liver, non-small cell lung, ovarian, pancreatic, prostate, skin, and thyroid cancers were identified as generally sensitive cancer types. Quantitative predicted sensitivity (as -log10 IC50) for each tumor class is shown in Fig. 6c.

### Model building and prediction using external platform

There are many available platforms and tools that implement advanced machine learning algorithms and hyper-parameter tuning to build the best predictive model. Some are specific to predicting drug sensitivity (Paccmann, CellminerCDB), while some are general purpose (Google AutoML, H2o.ai etc.). We used a Driverless AI platform from H2o.ai—the automatic machine learning platform, as it performs automated model tuning and facilitates derivation of suitable models capable of making predictions on independent datasets. As described in the Methods section, data were prepared and submitted to build the model. It ran a total of 199 tuning and 372 feature evolution models. The final model was a “bagged ensemble (pasting) of 1 LightGBM model across 3 folds”. Using the “Score on Another Dataset” option from the H2o interface, we obtained predicted IC50 values on the test set of 37 CCLE cell lines using the expression values of the same number of 1899 genes used in the training set. Table 1 shows the comparison of the prediction output on the 37 CCLE cell lines from H2o and RADR®.

**Table 1:** Comparative model performance on blind test data

Platform	Accuracy of sensitivity prediction within four fold of actual IC50 (%)	Accuracy of sensitivity prediction within two fold of actual IC50 (%)	Correlation coefficient	p value
RADR	86.49	54.05	0.7008	1.36e−06
H2o.ai	56.75	27.02	0.1313	0.4385

## Discussion

In summary, our key findings demonstrate that the machine learning derived signature from the best trained and tuned model—with a selected set of optimized parameters—predicted LP-184 sensitivity with > 85% accuracy. It is important to note that when using a relatively small training dataset i.e. NCI-60 screening data, it is challenging to derive a robust predictive gene signature. Here we have shown that our proprietary A.I. platform RADR® can be used effectively to derive a gene signature predictive of LP-184 response from NCI-60 data. This 16-gene signature appeared to be optimized for drug prediction outcomes on the entire (~ 1000 cell lines) CCLE dataset, based on the strong similarity of correlated gene pathways with the genes correlated to experimentally determined sensitivity. Toxicity of acylfulvenes requires intracellular reduction that is believed to be mainly mediated by PTGR1, which is highly expressed in a variety of cancer models and patient samples, and appears as a part of various prognostic or predictive models in cancer that are not necessarily drug-specific [37–40]. This suggests that acylfulvenes like LP-184 will be suited for patients exhibiting elevated tumor levels of PTGR1. PTGR1 being part of the LP-184-response signature helps to understand and consolidate the potential mechanism of action of the LP-184.

Here we have shown that LP-184, a next generation acylfulvene, exhibits broadly potent nanomolar toxicity in a spectrum of cancer types. LP-184 is a next-generation acylfulvene that is related to illudin S [14]. Acylfulvenes are a class of chemotherapeutic alkylating agents that are appealing due to their multifarious mechanisms of action involving diverse cellular components [41]. Acylfulvene-induced DNA-damage is reported to be exclusively repaired via the TC-NER pathway [5, 42], which is frequently deficient in cancers [43–45]. To date, clinical application of acylfulvenes has been limited, and it would therefore be useful to have methods of identifying patients who would respond well to drugs like LP-184 beforehand. However, measurement of PTGR1 alone, or any single gene, is not expected to adequately predict patient responsiveness. This is consistent with our measurements of PTGR1 and its correlation with LP-184 sensitivity in in vitro cytotoxicity assays. To better predict tumor response to this drug candidate, we sought to develop a more comprehensive, yet practically implementable signature, through the use of machine learning algorithms. Evaluation of individual signature genes for their potential connectivity with pathways and functions underlying LP-184 activity will also yield important insights into this compound’s mechanism of action as well as signature robustness.

Machine learning in cancer research has used data from tumor and/or patient features to predict clinical outcomes, ranging from general prognosis to response to a specific therapeutic option. This could be applied to improve conventional biomarker and diagnostic measures, and provide better informed, personalized patient-specific recommendations. Using biomarkers to inform chemotherapeutic choices has remained difficult, even in cases where the measured marker is known to be related to a drug target [46–48]. This may be partly due to cancer heterogeneity, which limits the inferences that can be drawn from using a single, or a small number, of features. The addition of other features may help overcome this limitation, but identifying and interpreting features that do so is a laborious process. Machine learning methods are capable of handling analysis of tens of thousands of features in numerous samples, giving an unbiased and high-throughput approach to the creation of a signature that is suitable for whole-genome omic data. In prediction of chemotherapeutic responses, drug IC50 values from various in vitro experiments provide a suitable format for modeling chemotherapeutic effects and developing predictive models through machine learning. We believe that machine learning will allow us to identify cancer subtypes that will be sensitive to compounds much faster and accelerate the drug development process.

Here we first attempted a variety of machine learning approaches, including SVM, ENR, RF, deep neural networks, that were evaluated alone or in combination. We found our initial training performed best when using a gene-correlation filter step, followed by initial selection with a random-forest method, and a final round of feature selection. This method generated the gene signature shown in Fig. 1b, and was kept in all of the following steps. This performed well when testing on a blind validation set from CCLE data (Fig. 1c). In addition to measuring prediction accuracy, we sought to compare the performance of this result with other proprietary artificial-intelligence methods. We accessed a state-of-the art H2o.ai artificial intelligence service [49], which evaluated the same training data. Although we were restricted in the number of genes that could be included and used, the training and test data was otherwise identical. This selected a “LightGBM” model as the most accurate with the adapted training set. We used their “Driverless AI” tool which automates the feature engineering, model building, visualization, and interpretability. The tool is supporting algorithms like Decision Tree, Follow the Regularized Leader (FTRL), Generalized Linear Models (GLM), Isolation Forest, LightGBM, RuleFit, TensorFlow & XGBoost. Based on the input data it automatically selects the suitable algorithm, tuning parameters, and outputs the best model. Compared with RADR®, there is substantially worse performance in predicting sensitivity with the blind CCLE test set. However, this method also selected PTGR1 as the most important feature in model predictions. Driverless AI feature importance is a measure of the contribution of an input variable to the overall predictions of the Driverless AI model. The final report provided detail about the relative feature importance, which is converted from feature importance. Collectively, this supported the selection of PTGR1 as the most important variable in our RADR® model, and that RADR® offered the best performance in accuracy on a blind test set.

Application of machine-learning to clinical cancer analysis has been promising but faces a number of challenges in practice. Depending on the training data available, there may be a limited sample quantity, which may also have substantial confounding properties—such as may occur when using a small number of observations from patients with diverse backgrounds. Training with certain predictive models also encounters challenges with regard to the type of prediction used. For example, predicting patient outcomes with a certain chemotherapeutic has been confounded with selection patterns that identify patients having generally better outcomes, regardless of drug or therapy. It has been found that in vitro measurement of drug sensitivity, by IC50 measurement, adequately reflects actual tumor sensitivity [50], and thus, tumor IC50 can provide a useful training target for models of chemotherapeutic efficacy. Often, biological data is not properly curated and cleaned. This makes it difficult to analyze the data effectively and appropriately. We are continuously building and using the RADR® database that is cleaned and properly tagged with metadata. Assuming sufficient data availability, cell lines may lack many of the unknown confounders that are found in patient data, and using IC50 to predict sensitivity will also avoid confounding drug response with overlapping clinical outcomes. Here we have exposed NCI-60 cell lines to LP-184 and experimentally determined each cell’s IC50.

To train a model predicting sensitivity from this data, we used the baseline transcriptome of cancer cells to train a model that selects which features (i.e., gene expression mRNA levels) together will determine the level of sensitivity to LP-184. Various features from cancer, including other forms of whole-genome omic measurements, have potential to be used as predictive markers. We compared other potential feature inputs, from DNA somatic mutations, copy-number variation, methylation, and others, for their capability to produce an accurate prediction model of LP-184 sensitivity in a test set, and found RNA expression to provide the greatest accuracy. This parallels what has been recently reported in cancer-sensitivity predictive model research [51, 52]. We found cancers of blood origin were highly resistant to LP-184 in general, and so removed these from training to leave 52 remaining solid tumor cell lines. Our training of approximately 23,000 transcripts resulted in a final signature of 16 genes that accurately predicted sensitivity across cancer classes. To validate the predictive power of this signature in our model with a blind test set, we used transcriptomes of the separate CCLE dataset in 37 CCLE cell lines for predictions, then experimentally validated their IC50. Model predictions were strongly correlated to LP-184 sensitivity, validating the prediction of drug response from mRNA expression.

Our model determined PTGR1 to be the most important feature for LP-184 sensitivity prediction, with PTGR1 alone having accounted for over half of all feature importance in the model (Fig. 1b). PTGR1 has a well-established critical role to mediate intracellular activation of acylfulvenes. This supports that the gene expression features with important mechanistic roles can be selected from the 16-gene model built with expression and drug sensitivity data. In numerous cancer types, PTGR1 expression is elevated [53]. Studies in a variety of cancer types have reported PTGR1 is involved in progression and malignancy [36, 54]. In this context it is thus considered desirable to have PTGR1-based sensitivity for enhancing cancer drug selectivity and targeting aggressive cancers. Despite the major importance, PTGR1 appears to be necessary, but not sufficient, for LP-184 sensitivity—PTGR1 levels alone are not capable of providing high-confidence predictions of sensitivity. However, with the 16-gene model, we are able to compensate for this limitation and provide an approach that could be applied to making informed clinical decisions with a new type of acylfulvene. In general, the bioactivation and anti-tumor activity of the acylfulvene class of prodrugs appears to be based on the enzymatic action of PTGR1. Thus, LP-184 sensitivity is likely to be dependent upon the oxidoreductase activity of PTGR1, along with other factors. RADR® has demonstrated hypothesis-free identification of biomarkers with biological relevance and statistical rigor with highest possible prediction accuracy. Moreover, at least one gene, i.e. PTGR1, the top-weighted gene from the final 16 predictive signature, is functionally associated with acylfulvene-specific induction of bioactivation and is in agreement with the perceived mechanism of action of LP-184. These preliminary biomarker analyses will be further validated using LP-184 sensitivity and gene expression data derived from additional independent advanced tumor models.

We looked into the relationship of the predictive model genes to cancer biology and the mechanism of action of acylfulvene chemotherapeutics. A number of signature genes (Table 2) had a known role or link to DNA damage repair and cancer progression, which possibly explains why they were selected by our algorithmic model. In some cases, signature gene expression is associated with cancer progression and poor outcomes, but also correlated with LP-184 sensitivity. We also sought to analyze functional relations with enrichment analysis methods. However, a signature produced by machine learning is not necessarily expected to reflect a biological process in traditional enrichment analysis, particularly when the signature itself contains a small number of genes. This is also a consequence of machine learning methods being designed to select non-redundant features, which is antagonistic to selection of genes that have highly correlated expression—and therefore antagonistic to selecting genes in the same pathway. Given that XGBoost is a method that avoids the problem of collinearity, it is not surprising we are not able to see a clear enrichment pattern with the 16 gene signature alone. XGBoost is being used more widely in recent years as an algorithm of choice for generating drug response predictions [55, 56]. Nevertheless, the previously reported functions of these signature genes support a functional association with mechanisms of acylfulvene toxicity.

**Table 2:**
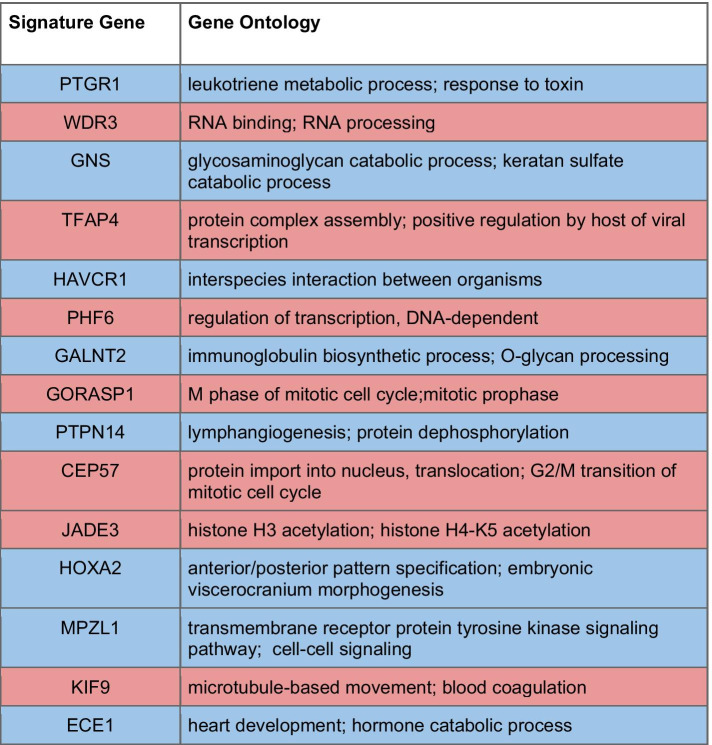
Links between signature genes and cancer and DNA damage repair

The 16 signature genes predicting LP-184 sensitivity are listed in this table in order of their weighted importance, colored blue to indicate a positive correlation between the expression of the gene and LP-184 sensitivity, and red for negative correlations. Annotated functions show the gene ontology annotation

We performed an enrichment analysis using the genes that strongly correlated to actual LP-184 sensitivity and to predicted sensitivity, in the NCI-60 and CCLE databases, respectively. Firstly, this approach has the potential to support the validity of our predictions on the total 1000 cell lines in the CCLE. It is reasonable to expect that if predictions of sensitivity are accurate, we will see similar patterns of gene enrichment. Secondly, this will also be informative on the potential mechanisms of sensitivity; the enrichment analysis when using the top 100 positive and negative genes correlated to sensitivity could reflect patterns of gene expression that contribute to LP-184 response. After performing enrichment analysis in this manner, we found that the top correlated genes to LP-184 actual or predicted sensitivity select genes in the same pathways. There was an extremely high degree of overlap between the number of genes in the same pathway. This is not necessarily expected when considering that the cell lines used do not generally have highly correlated transcriptomes or functional pathways. Together, this further supports that the predictions in the CCLE 1000 cell lines were accurate, and also suggests that the model predictions are capable of selecting functional patterns from transcriptomes.

To determine biological pathway associations with LP-184 sensitivity, we instead sought to analyze the genes which were correlated to LP-184 sensitivity or predicted sensitivity from our model. Not only do we find a variety of pathways significantly enriched (Fig. 5), but we also observed nearly equal representation of these pathways in both groups. This suggests that these correlated genes that in turn narrow down to signature genes, are capable of predicting actual functional processes, as opposed to sensitivity being a byproduct of random chance or noise in transcription. It also suggests that our model is capable of identifying drug-specific biological functions that would not be predictable by cell-type or cancer-type classification alone. The different cell types predicted to be sensitive have substantial differences in transcriptomes and phenotypic characteristics yet have a hidden signature that can be revealed by machine learning models to predict phenotypes of clinical value. This points to the value of using a machine learning method in deriving a gene signature. Traditional methods of expression or enrichment analysis would not suffice to make highly accurate predictions of drug candidates such as LP-184, nor would attention to a few genes known from individual studies be a likely substitute.

**Fig. 7:**
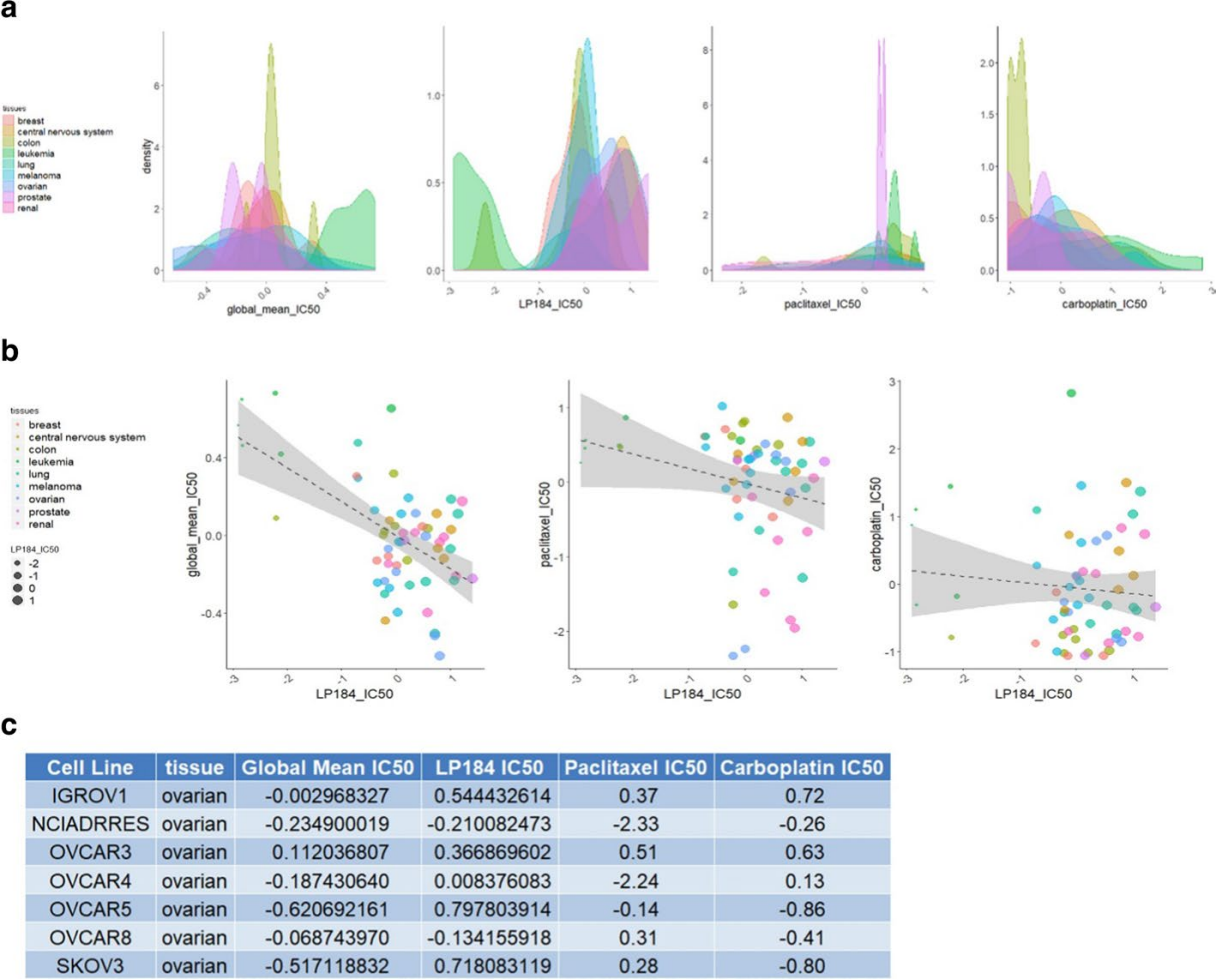
Comparison of LP184 drug sensitivity profile with other drugs. **a** The frequency of different sensitivity levels is shown for each tissue for different drug groups. The IC50 is shown as a z-score, with the higher score indicating greater sensitivity of the corresponding cell lines or tissues. The global mean IC50 refers to the average z-score of each of the > 20,000 compounds in the NCI-60 dataset. **b** Pairwise comparisons of drug sensitivity in individual cell lines. For distinguishing points of interest, the point size is proportional to LP184 drug sensitivity. LP-184 shows higher z-scores for ovarian cancer, compared to the global average, and, in some lines, to individual drugs used clinically in ovarian cancer. **c** Table of IC50 z-score values for LP-184, paclitaxel, and carboplatin drugs, and the average of > 20,000 compounds, in 7 ovarian cancer lines from the NCI-60 panel

We found that some cancers are more frequently predicted to be sensitive to LP-184, with our model predicting that bladder, esophageal, head and neck, kidney, liver, non-small cell lung, ovarian, pancreatic, prostate, skin, and thyroid cancers will be responsive more often. However, a precise determination of suitability can be made by measuring the expression of the 16 genes, as there are exceptions in each cancer. These analyses might help find new frontline options for cancers that lack effective chemotherapeutic options or other treatments. Though there are many potential cancer indications in which LP-184 might be explored as a frontline option, our experimental data from NCI-60 lines indicated that LP-184 has appreciable activity in ovarian cancers, which do relatively poorly in sensitivity to more than 20,000 other compounds tested in the NCI-60 database (Fig. 7ab). Among ovarian cancer in these data, there is a higher proportion with an LP-184 z-score over 0.5, that indicates high sensitivity (Fig. 7a). As shown in the table in Fig. 7, LP-184 ranked the highest in sensitivity in 3 of the 7 ovarian cancer cell lines. In particular, in OVCAR5 and SKOV3 (known as a paclitaxel-resistant line) there was a high sensitivity to LP-184, but they have a below average response to other compounds. Given the need for new frontline treatments in ovarian cancer and the sensitivity shown here, clinical development of LP-184 in ovarian cancer would be worth investigating. However, other cancer types may also reveal this characteristic when more data are explored. Interestingly, LP-184 showed a distinctive wide range of sensitivity with some cancer types (Fig. 7b). This indicates large potential uses, and also a need to have a more rigorous method to select cancers for treatment than using cancer type alone. Ultimately, clinical use of LP-184 would ultimately be best served by a prognostic measurement that uses expression data to stratify patients as potential responders, in ovarian, and other cancer types.

## Conclusions

Our key findings demonstrate that the machine-learning-derived signature from the best trained and tuned XGBoost model can predict LP-184 sensitivity in solid tumors with > 85% accuracy. This was validated on a blind training set of cell lines selected from the CCLE, and when performing predictions on the 1000 available CCLE lines, was able to make predictions that appear highly similar in functional gene expression patterns. To utilize this signature, only 16 genes (Fig. 1b) are necessary to be able to predict sensitivity in at least 23 cancer types of distinct origin. The gene signature generated by our model includes genes critical for defining tumor specificity of LP-184. This suggests that the signature is suitable to be applied in the clinical development of LP-184 as it has captured the mechanism of PTGR1 involvement in LP-184 activity.

## Methods

In this section, the data acquisition and processing steps are described in detail.

### Data acquisition

We have used NCI CellMinerCDB platform to download the microarray log2 expression data of NCI-60 and CCLE (Cancer Cell Line Encyclopedia). The LP-184 IC50 values were derived by NCI-60 screening of the compound. From the CCLE set of cell lines, we selected 37 cell lines as the blind testing set in which we generated LP-184 IC_50_ values from dose response curves using the CellTiter Fluor assay method. Expression data from CCLE were used to predict the sensitivity (IC_50_) of CCLE cell lines to LP-184. The downloaded files from CellMinerCDB platform are (1) data_nci60_xai.txt and (2) data_ccle_exp.txt.

### Data processing

#### Training data

The LP-184 sensitivity and gene expression data were available for a total of 58 cell lines. Of these, 6 blood cancer cell lines were excluded from solid tumor specific data analyses. The resulting data served as training data from which the LP-184 response gene signature was derived. There were a total of 23,059 genes and 52 cell lines in the training data.

#### Blind testing data

37 cell lines from CCLE were selected to be tested at REPROCELL USA, Inc. for LP-184 activity in order to derive drug sensitivity (IC_50_) values. The blind testing data were generated by extracting expression data for only those 37 cell lines from CCLE. There were a total of 19,851 genes and 37 cell lines in the blind testing data.

#### CCLE data

In order to identify potential sensitive and resistant tumor types, we have used CCLE cell line gene expression data. In the downloaded data, there were a total 36 common cell lines that are also a part of NCI-60 data (covered in the training model). Hence, we removed those cell lines. There were a total of 19,851 genes and 1000 cell lines in the CCLE data, that we used to identify potential indications for LP-184.

#### Cell growth measurements

Human tumor cell lines were grown in RPMI 1640 medium containing 5% fetal bovine serum and 2 mM L-glutamine. Cells were inoculated into 96 well microtiter plates in 100 μL at plating densities ranging from 5,000 to 40,000 cells/well depending on the doubling time of individual cell lines. After cell inoculation, the microtiter plates were incubated at 37° C, 5% CO2, 95% air and 100% relative humidity for 24 h prior to addition of LP-184. After 24 h, LP-184 from a 10 mM stock solution in DMSO was added in triplicate wells to the desired test concentrations in the range of 1 nM to 10 uM. Following drug addition, the plates were incubated for an additional 48 h at 37 °C, 5% CO2, 95% air, and 100% relative humidity. The assay was terminated by the addition of cold TCA. Cells were fixed in situ by the gentle addition of 50 μl of cold 50% (w/v) TCA (final concentration, 10% TCA) and incubated for 60 min at 4 °C. The supernatant was discarded, and the plates were washed five times with tap water and air dried. Sulforhodamine B (SRB) solution (100 μl) at 0.4% (w/v) in 1% acetic acid was added to each well, and plates are incubated for 10 min at room temperature. After staining, unbound dye was removed by washing five times with 1% acetic acid and the plates were air dried. Bound stain was subsequently solubilized with 10 mM trizma base, and the absorbance was read on an automated plate reader at a wavelength of 515 nm. Using the absorbance measurements, Inhibitory Concentration of 50% (IC50), which is the drug concentration resulting in a 50% reduction in the net protein increase (as measured by SRB staining) in control cells during the drug incubation, was calculated.

### Signature derivation process

We have used Lantern’s proprietary RADR® platform in order to step by step identify and reduce the statistically significant and biologically relevant genes and finally derive both robust and manageable biomarkers. The platform first identifies the genes that are significantly (*p value* <  = 0.05) correlated both positively and negatively with LP-184 IC_50_ values using a Pearson correlation method. Pathway analysis (using Pathcards database covering 3840 pathways) and gene network analysis (using Pathway commons database covering 1,546,602 interactions) were performed separately on the set of correlated genes in order to retain the genes that appear to be biologically relevant. In pathway analysis, the known drug target pathway is used to narrow down the correlated genes and to output only the genes that are directly or indirectly related to the drug's target pathway. The gene network analysis outputs the network of genes that are connected with correlated genes. Lastly, another inductive learning algorithm, termed the ‘Relief algorithm’ ranks and assigns weights to these filtered genes based on drug sensitivity (IC_50_). The highly ranked genes (top 100) are used in the subsequent prediction module. The prediction module runs the ‘Boruta’ feature selection algorithm on these ranked genes to obtain the significant gene features (*p value* cutoff = 0.05). In order to reduce the set of potential response predictor genes even further, based on the ranking, genes get eliminated one by one, and the model gets built to compare the prediction accuracy on the test set. This step performs 5-time repeated tenfold cross validation using RMSE as the validation metric. Prediction accuracy is calculated by dividing the number of cell lines with predicted LP-184 IC50 within two or four fold from actual LP-184 IC50, by the total number of cell lines in the blind set and expressed as a fraction ranging from 0 to 1. Performance is evaluated based on the sum of the ‘Two-Fold’ and ‘Four-Fold’ testing accuracy during the iterative loop. The final model and signature set chosen is the one with the highest accuracy sum. The 16 gene signature was selected as the best signature. Flow chart of the entire process is given in the Additional file 1: Figure S1.

### Testing of signature on blind test data

We used the tuned model and expression of only the above identified 16 signature genes to predict the IC_50_ of blind test data having 37 cell lines described in the “Blind testing data” section of the data processing step. The predicted values were compared with the actual IC_50_ values in order to evaluate the signature performance.

### Prediction on CCLE data

We have used the expression levels of 16 signature genes on the entire CCLE data described in the “CCLE data” section of the data processing step and tuned the model to predict LP-184 sensitivity (IC_50_ values). In order to evaluate the prediction, we compared the outcome with NCI-60 data for which the LP-184 sensitivity is known.

All the figures except the heatmap were generated using the ggplot package in R. The heatmap was generated using the pheatmap package in R. For heatmap hierarchical clustering, we used “euclidean” as a distance measure and “complete” as a clustering method. The correlation in the scatter plot was calculated using the Pearson correlation method.

### External platform data preparation and processing (H2o.ai)

We have used the training data having exactly the same number of cell lines i.e. 52 NCI-60 solid tumor cell lines, and only highly correlated NCI-60 genes that are commonly present in the 37 CCLE cell line data i.e. blind data. (We were unable to use all the 23,059 genes due to the platform limitation, which requires <  = 10,000 feature). In order to select the highly correlated genes, we first performed the Pearson correlation (between the gene expression and IC50 from 52 cell lines) and selected the genes that are statistically significant (p value <  = 0.05) followed by matching the genes that are present in the blind testing data set. In the resulting training data, there were 52 cell lines and 1899 genes. The Experiment was conducted with train data and default regression model settings from the H2o platform.

## Supplementary Information

**Additional file 1: Figure S1**. Flow chart describing statistical methodology.

**Additional file 2: Figure S2**. Comparison of the different model accuracy on blind test data.

**Additional file 3: Table S1**. List of tumor types covered in the test set of 1000 CCLE cell lines.

**Additional file 4: Table S2**. List of 52 cell lines with experimental IC50 (Molar).

**Additional file 5: Table S3**. Enrichment analysis of top 200 highly correlated genes from 58 NCI-60 cell lines using experimental IC50.

**Additional file 6: Table S4**. Enrichment analysis of top 200 highly correlated genes from 1000 CCLE data using predicted IC50.

## Data Availability

The datasets generated and/or analyzed during the current study are available in the CellMinerCDB repository, available through https://discover.nci.nih.gov/cellminercdb/ [31].

## Abbreviations

CCLE: Cancer Cell Line Encyclopedia
NCI: National Cancer Institute
RADR®: Response Algorithm for Drug positioning and Rescue
AF: Acylfulvene
DGE: Differential Gene Expression
MFA: Multiple Factor Analysis
SVM: Support Vector Machines
ENR: Elastic Net Regression
RF: Random Forest
RMSE: Root Mean Squared Error
FDR: False Discovery Rate
