# Supplementary material for: A machine learning-based gene signature of response to the novel alkylating agent LP-184 distinguishes its potential tumor indications
Source: BMC Bioinformatics. 2021 Mar 2;22:102. doi: 10.1186/s12859-021-04040-8 (PMC7923321; doi:10.1186/s12859-021-04040-8)

Manuscript Title:- A machine learning-based gene signature of response to the novel alkylating agent LP-184 distinguishes its potential tumor indications

Authors:- Umesh Kathad1*, Aditya Kulkarni1, Joseph Ryan McDermott1, Jordan Wegner1, Peter Carr1, Neha Biyani1, Rama Modali2, Jean-Philippe Richard2, Panna Sharma1, Kishor Bhatia1

1: Lantern Pharma, Inc. 1920 McKinney Ave, 7th floor, Dallas TX 75201 USA

2: REPROCELL USA Inc. 9000 Virginia Manor Rd, Ste 207, Beltsville MD 20705 USA

*Corresponding author, email: [umesh@lanternpharma.com](mailto:umesh@lanternpharma.com)

Supplemental Figure 1. Flow chart describing statistical methodology


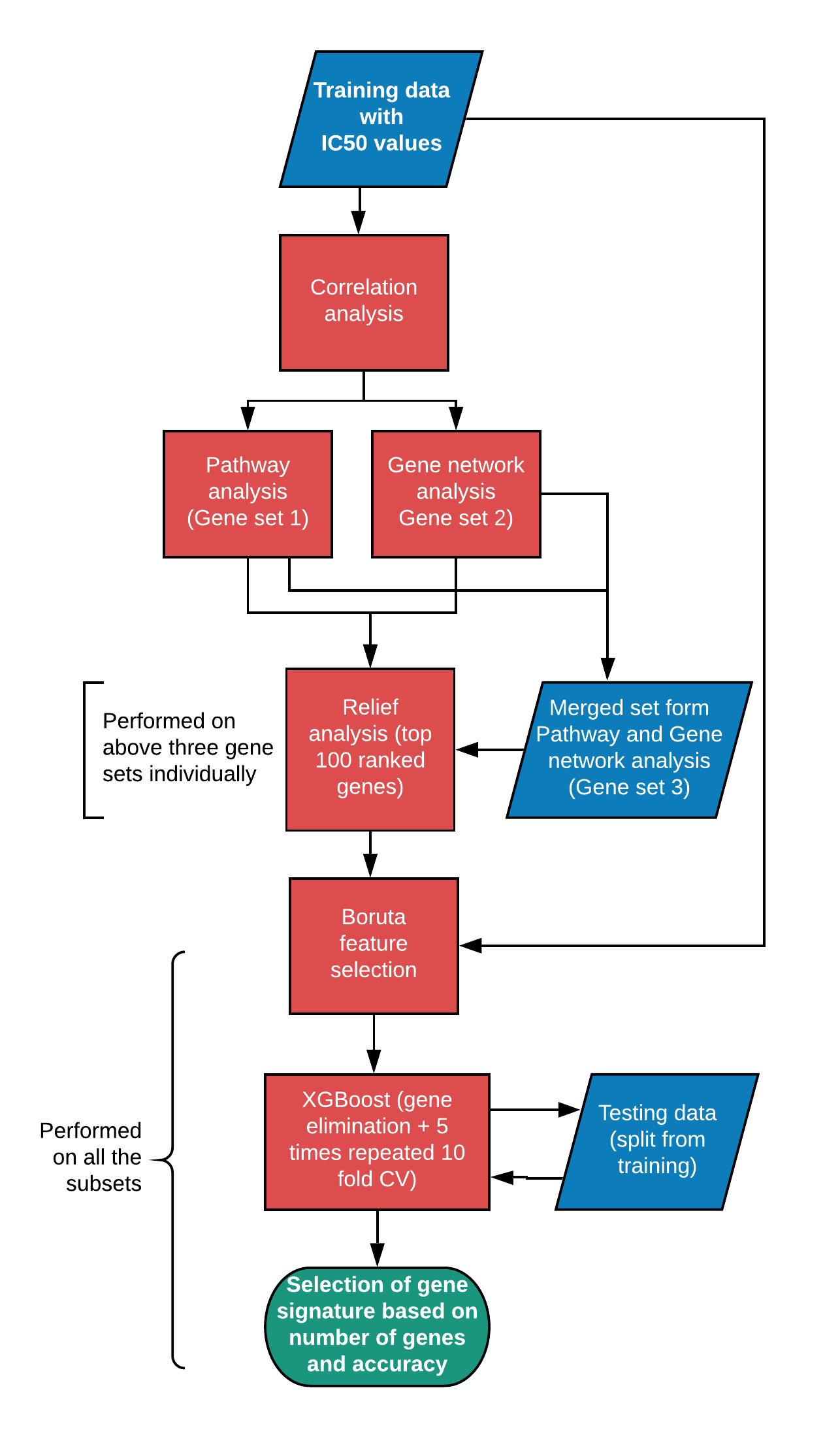

Supplement: Supplementary file 1 — Additional file 1: Figure S1. Flow chart describing statistical methodology. [file 12859_2021_4040_MOESM1_ESM.docx]
