# Supplementary material for: A machine learning-based gene signature of response to the novel alkylating agent LP-184 distinguishes its potential tumor indications
Source: BMC Bioinformatics. 2021 Mar 2;22:102. doi: 10.1186/s12859-021-04040-8 (PMC7923321; doi:10.1186/s12859-021-04040-8)
Supplement: Supplementary file 2 — Additional file 2: Figure S2. Comparison of the different model accuracy on blind test data. [file 12859_2021_4040_MOESM2_ESM.docx]

Manuscript Title:- A machine learning-based gene signature of response to the novel alkylating agent LP-184 distinguishes its potential tumor indications

Authors:- Umesh Kathad1*, Aditya Kulkarni1, Joseph Ryan McDermott1, Jordan Wegner1, Peter Carr1, Neha Biyani1, Rama Modali2, Jean-Philippe Richard2, Panna Sharma1, Kishor Bhatia1

1: Lantern Pharma, Inc. 1920 McKinney Ave, 7th floor, Dallas TX 75201 USA

2: REPROCELL USA Inc. 9000 Virginia Manor Rd, Ste 207, Beltsville MD 20705 USA

*Corresponding author, email: [umesh@lanternpharma.com](mailto:umesh@lanternpharma.com)

Supplemental Figure 2. Comparison of the different model accuracy on blind test data


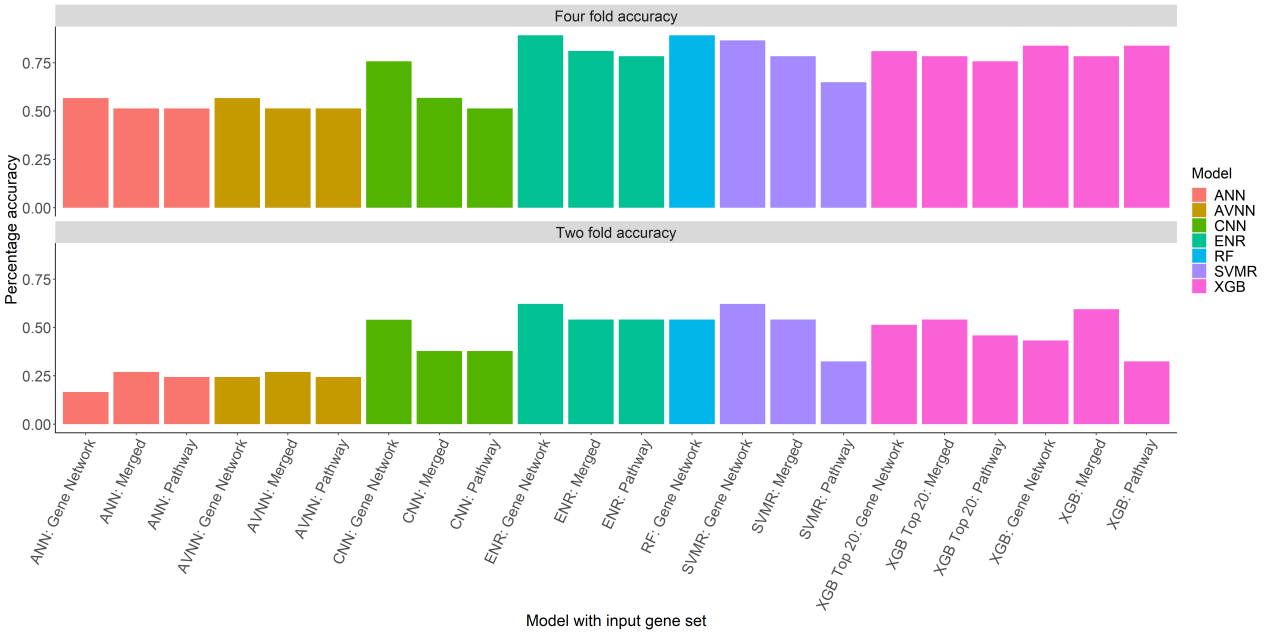


ANN = Artificial Neural Network

AVNN = [Averaged Neural Network](https://stackoverflow.com/questions/52222976/is-an-averaged-neural-network-avnnet-the-average-from-all-iterations)

CNN = [Convolutional Neural Network](https://en.wikipedia.org/wiki/Convolutional_neural_network)

[ENR = Elastic Net Regression](https://en.wikipedia.org/wiki/Convolutional_neural_network)

[RF = Random Forest](https://en.wikipedia.org/wiki/Convolutional_neural_network)

[SVMR = Support Vector Machine Regression](https://en.wikipedia.org/wiki/Convolutional_neural_network)

[XGB = XGBoost](https://en.wikipedia.org/wiki/Convolutional_neural_network)
